# A 12-month follow-up of the immune response to SARS-CoV-2 primary vaccination: evidence from a real-world study

**DOI:** 10.3389/fimmu.2023.1272119

**Published:** 2023-11-20

**Authors:** Giorgio Fedele, Ilaria Schiavoni, Filippo Trentini, Pasqualina Leone, Eleonora Olivetta, Alessandra Fallucca, Stefano Fiore, Angela Di Martino, Sergio Abrignani, Vincenzo Baldo, Tatjana Baldovin, Alessandra Bandera, Pierangelo Clerici, Massimo De Paschale, Fabiana Diaco, Alexander Domnich, Francesca Fortunato, Irene Giberti, Andrea Gori, Renata Grifantini, Tiziana Lazzarotto, Vittorio Lodi, Claudio Maria Mastroianni, Rosa Prato, Vincenzo Restivo, Francesco Vitale, Silvio Brusaferro, Stefano Merler, Anna Teresa Palamara, Paola Stefanelli, the Study Group for the Immunological Monitoring post Covid19 vaccination

**Affiliations:** ^1^ Department of Infectious Diseases, Istituto Superiore di Sanità, Rome, Italy; ^2^ Center for Health Emergencies, Bruno Kessler Foundation, Trento, Italy; ^3^ Dondena Centre for Research on Social Dynamics and Public Policy, Bocconi University, Milan, Italy; ^4^ National Center for Global Health, Istituto Superiore di Sanità, Rome, Italy; ^5^ Department of Health Promotion, Mother and Child Care, Internal Medicine and Medical Specialties “G. D’Alessandro”, University of Palermo, Palermo, Italy; ^6^ INGM, Istituto Nazionale Genetica Molecolare “Romeo ed Enrica Invernizzi”, Milan, Italy; ^7^ Department of Clinical Sciences & Community Health, University of Milan, Milan, Italy; ^8^ Laboratory of Hygiene and Applied Microbiology, Hygiene and Public Health Unit, Department of Cardiac Thoracic and Vascular Sciences and Public Health, University of Padova, Padova, Italy; ^9^ Infectious Diseases Unit, Foundation IRCCS Ca’ Granda Ospedale Maggiore Policlinico, Milan, Italy; ^10^ Centre for Multidisciplinary Research in Health Science (MACH), University of Milano, Milan, Italy; ^11^ Microbiology Unit, Azienda Socio Sanitaria Territoriale (ASST) Ovest Milanese, Milan, Italy; ^12^ Department of Molecular Medicine, AOU Policlinico Umberto I, Sapienza University, Rome, Italy; ^13^ IRCCS Ospedale Policlinico San Martino Genova, and Department of Health Sciences, University of Genoa, Genoa, Italy; ^14^ Hygiene Unit, Policlinico Riuniti Foggia Hospital, Department of Medical and Surgical Sciences, University of Foggia, Foggia, Italy; ^15^ II Division of Infectious Diseases, "Luigi Sacco" Hospital, ASST Fatebenefratelli Sacco, Milan, Italy; ^16^ Microbiology Unit, IRCCS Azienda Ospedaliero-Universitaria di Bologna, Bologna, Italy; ^17^ Section of Microbiology, Department of Medical and Surgical Sciences, University of Bologna, Bologna, Italy; ^18^ Occupational Health Unit, IRCCS Azienda Ospedaliero-Universitaria di Bologna, Bologna, Italy; ^19^ Department of Public Health and Infectious Disease, AOU Policlinico Umberto I, Sapienza University, Rome, Italy; ^20^ Istituto Superiore di Sanità, Rome, Italy

**Keywords:** SARS-CoV-2, vaccines, immune response, serology, T-cell

## Abstract

A real-world population-based longitudinal study, aimed at determining the magnitude and duration of immunity induced by different types of vaccines against COVID-19, started in 2021 by enrolling a cohort of 2,497 individuals at time of their first vaccination. The study cohort included both healthy adults aged ≤65 years and elderly subjects aged >65 years with two or more co-morbidities. Here, patterns of anti-SARS-CoV-2 humoral and cell-mediated specific immune response, assessed on 1,182 remaining subjects, at 6 (T6) and 12 months (T12) after the first vaccine dose, are described. At T12 median anti-Spike IgG antibody levels were increased compared to T6. The determinants of increased anti-Spike IgG were the receipt of a third vaccine dose between T6 and T12 and being positive for anti-Nucleocapside IgG at T12, a marker of recent infection, while age had no significant effect. The capacity of T12 sera to neutralize *in vitro* the ancestral B strain and the Omicron BA.5 variant was assessed in a subgroup of vaccinated subjects. A correlation between anti-S IgG levels and sera neutralizing capacity was identified and higher neutralizing capacity was evident in healthy adults compared to frail elderly subjects and in those who were positive for anti-Nucleocapside IgG at T12. Remarkably, one third of T12 sera from anti-Nucleocapside IgG negative older individuals were unable to neutralize the BA.5 variant strain. Finally, the evaluation of T-cell mediated immunity showed that most analysed subjects, independently from age and comorbidity, displayed Spike-specific responses with a high degree of polyfunctionality, especially in the CD8 compartment. In conclusion, vaccinated subjects had high levels of circulating antibodies against SARS-CoV-2 Spike protein 12 months after the primary vaccination, which increased as compared to T6. The enhancing effect could be attributable to the administration of a third vaccine dose but also to the occurrence of breakthrough infection. Older individuals, especially those who were anti-Nucleocapside IgG negative, displayed an impaired capacity to neutralize the BA.5 variant strain. Spike specific T-cell responses, able to sustain immunity and maintain the ability to fight the infection, were present in most of older and younger subjects assayed at T12.

## Introduction

The emergence of SARS-CoV-2 and its rapid global transmission has prompted an urgent need for effective vaccines to control the COVID-19 pandemic. The immune response to SARS-CoV-2 vaccines involves a complex interplay of innate and adaptive immunity, orchestrated by various immune cells, cytokines, and antibodies. Understanding the intricate dynamics of this response is essential for assessing vaccine efficacy, durability of protection, and the potential for emerging variants to escape vaccine-induced immunity. As the pandemic continues to evolve, the development and deployment of effective vaccines have become crucial in the fight against SARS-CoV-2. Knowledge of the immune response to vaccination can inform the development of future vaccine strategies, including booster doses and groups at risk.

Several studies have analysed the temporal trends of SARS-CoV-2 specific antibodies induced by vaccination, and the correlation between immunoglobulin (Ig) G levels and neutralizing activity. Overall, mRNA vaccines have been shown to be highly effective in the first months after vaccination against symptomatic COVID-19 (1–5). Nevertheless, humoral immunity gradually declines few months after receiving the primary vaccine schedule. Six to eight months after vaccination, Spike-specific antibody titers and neutralizing antibody activity were significantly lower than the peak titers (6, 7). However, a decline in vaccine-induced protection against hospitalization and death for COVID-19 after 6 months from the second dose of vaccine has not been documented, suggesting that cellular immunity could have a crucial protective role, restricting viral spread and resolving infection when antibodies wane (8). T-cell responsiveness against SARS-CoV-2 was found to be present in patients treated with immunosuppressive agents with no serological response to mRNA vaccines (9, 10). To date it is postulated that T-cell responses are effective in preventing COVID-19 infection, or at least severe disease, and, as they are predominantly directed toward epitopes encompassing conserved peptides, can respond to SARS-CoV-2 variants (11–13).

Understanding post-vaccination antibody persistence is complicated by patient-dependent factors and characteristics. Measurements of antibody responses to vaccination against SARS-CoV-2 vary greatly based on age, gender, pathological conditions, current therapies and pre-existing level of infection-induced antibodies (2, 14–16). Conflicting data about the determinants influencing the immune response to vaccination are available in the literature and need further investigation. Furthermore, the rapid succession of virus variants has made it difficult to identify an antibody titre able to confer protection against COVID-19. The correlates of protection of SARS-CoV-2 infection have not yet been unequivocally defined, although a much higher antibody titre is thought to be required to neutralize the Omicron lineages and sublineages than the ancestral virus (Wild-type) (17, 18). The estimate of a correlate of protection could allow the identification of people low responders who do not seroconvert effectively, to plan future targeted vaccination boosters.

Data presented here stemmed from a project aimed at evaluating magnitude and duration of immunity induced by anti COVID-19 vaccination (2). Building upon the findings from our previous study, here we investigated the changes in anti-SARS-CoV-2 specific immune responses between 6 and 12 months after the first vaccination.

Overall, we provide a comprehensive overview of various aspects of the immune response to SARS-CoV-2 vaccines in a changing evolutionary landscape, marked by the appearance of the Omicron lineages and sublineages.

## Methods

### Study design and population

A real-world, population-based longitudinal monitoring of immune responses to SARS-CoV-2 vaccines was conducted, enrolling 2497 individuals at the time of their first SARS-CoV-2 vaccination (2). Two cohorts, adults ≤65 years of age and frail subjects >65 years of age with at least two co-morbidities associated with increased risk of severe COVID-19 [listed in (2)] were enrolled in eight collaborating centres from seven Italian regions. Venous blood withdrawals for serum preparation and peripheral blood mononuclear cells (PBMCs) isolation were planned at first vaccination, one month after the completion of the primary vaccine series, 6 months after the first vaccine dose, and 12 months after the first vaccine dose. During the 12 months follow-up period, most of the subjects dropped out of the study. Hence, the final study sample consisted of 1182 persons.

### Serum preparation and storage

Serum samples were obtained by collecting peripheral blood (5 ml) in Serum Separator Tubes (BD Diagnostic Systems, Franklin Lakes, NJ, USA) and subsequent centrifugation at 1600 rpm for 10 min at room temperature. Two aliquots of purified serum for each donor were transferred to 2ml polypropylene, screw cap cryo tubes (Nunc™, Thermofisher Scientific, Waltham, MA USA). Aliquots were immediately frozen at -20°C and thereafter stored at -80°C. Frozen sera were shipped in dry ice to the Department of Infectious Diseases at Istituto Superiore di Sanità (DMI, ISS), following biosafety shipment condition. Upon arrival serum samples were immediately stored at -80°C.

### SARS-CoV-2 IgG immunoassays

Levels of anti-SARS-CoV-2 Spike IgG were assessed centrally at DMI, ISS. The DiaSorin Liaison SARS-CoV-2 trimeric Spike (S) IgG chemiluminescence immunoassay (CLIA) assay was used on the LIAISON® XL chemiluminescence analyzer (DiaSorin, Saluggia, VC, Italy). The assay is able to specifically detect IgG directed against the native trimeric form of the Spike protein. The assay range is up to 2080 Binding Antibody Units (BAU/mL). According to manufacturer’s instructions, values ≥ 33.8 BAU/mL were interpreted as positive. If the results were above the assay range, samples were automatically diluted 1/20 and testing was repeated.

Anti-Nucleocapsid (N) IgG were measured by different immunoassys at each of the collaborating centres. Immunoassays used were the anti-N IgG Elecsys (Roche Diagnostics, Monza, Italy), the anti-N IgG iFlash (Pantec, Torino, Italy) and anti-N IgG Architect (Abbott Diagnostics, Chicago, IL, USA). For each assay data were interpreted according to manufacturers’ instructions.

### SARS-CoV-2 neutralizing antibody assays

A subgroup of 80 enrolled subjects was selected by simple randomization to perform SARS-CoV-2 neutralizing assays. A SARS-CoV-2 strain belonging to the B lineage (B.1, WT), considered as a reference strain in this study, and a variant strain belonging to the Omicron BA.5 lineage, were incubated with two-fold serial dilutions of serum samples; dilutions ranged from 1:8 to 1:1024. Sera were diluted in E MEM culture medium (Sigma Aldrich, Merck Life Science, Milan, Italy) supplemented with 1X penicillin/streptomycin (Corning, Glendale, AZ, USA) and 2% foetal bovine serum (Corning) in 96-well plates (Sarsted, Nümbrecht, Germany). Virus (100 TCID50) was mixed with serum and incubated at 37°C for 1 hour. Then, 10,000 cells were added to each well and incubated at 37°C for 5 days. The neutralization titer was calculated and expressed as microneutralization titer 50 (MNT50), i.e., the serum dilution capable of reducing the cytopathic effect to 50%. Due to technical issues, we were unable to calculate titers from a few sera and final analyses was performed on a total of 73 subjects.

### Assessment of SARS CoV-2 spike-specific T-cell response

A subgroup of 120 enrolled individuals were selected by simple randomization for assessment of T-cell responses at the T12 time-point. Briefly, peripheral blood mononuclear cells (PBMCs) were purified at the collaborating centers from venous heparinized blood samples by density gradient and immediately frozen at -80°C. Samples were then shipped to DMI, ISS, and stored in liquid nitrogen. After thawing, Spike protein-specific T-cell responses were measured by stimulating patients’ PBMCs with a pool of overlapping peptides covering the immunodominant domains of the wild type Spike protein (Miltenyi, Bergisch Gladbach, Germany), used at a final concentration of 0.6 nmol of each peptide/ml. For each sample, negative and positive controls tube were prepared with unstimulated PBMCs or staphylococcal enterotoxin B (SEB) stimulated cells (100 ng/ml; Sigma-Aldrich), respectively. Three hours after stimulation Brefeldin A (3 μg/ml) was added to prevent cytokine secretion. After overnight stimulation, cells were incubated with Live/Dead fixable violet dead cell stain kit used to exclude dead cells from the analyses (Thermo Fisher Scientific). Cells were then fixed and permeabilized using Cytofix/Cytoperm Fixation/Permeabilization Solution Kit (ThermoFisher) and stained with a predetermined optimal concentration of fluorochrome-conjugated Abs: anti-CD3-APC-H7, anti-IL-2-FITC, anti-TNF-α PE-Cy7 (all from BD Biosciences, Franklin Lakes, NJ, USA), anti-IFN-γ-PerCP-Cy5.5 (both from Biolegend, San Diego, CA, USA), anti-CD8-APC (eBiosciences, Thermo Fisher Scientific). Cells were then acquired by a Gallios Flow Cytometer (Beckman Coulter, Indianapolis, IN, USA) and data analyzed with Kaluza Analysis software (Beckman Coulter). Frequencies of cytokine producing cells were calculated after subtraction of cytokine positive cells in the unstimulated sample, i.e., negative control tube. Due to technical issues, vital cell recovery was unsatisfying in several samples and final analyses were performed on peripheral blood mononuclear cells (PBMCs) from 103 subjects.

### Statistical analysis

Using a log-linear regression model, it was investigated the association between the geometric mean anti- SARS-CoV-2 trimeric S IgG titers at T12 and available covariates of interest: a dichotomous variable that indicates whether study participants received the third dose, anti-N IgG status at T12, age group, sex, the log-transformed anti- SARS-CoV-2 trimeric S IgG titers at T12, and a variable indicating whether the study participants received mRNA vaccines in the primary cycle. We considered the log-transformation of the dependent variable in the model due to the skewness of the distribution of the anti- SARS-CoV-2 trimeric S IgG titers.

The Spearman correlation coefficient and correlation test were conducted to analyse the correlation between anti-SARS-CoV-2 trimeric S IgG titers at T12 with neutralization and T-cell response. The relative P-values were compared across different characteristics of study participants (sex, age group and IgG N status). Differences in neutralization between female and male study participants, between healthy subjects under 65 years of age and frail elderly over 65 years of age, and between study participants with positive or negative anti-N IgG at T12 were assessed through the Mann-Whitney nonparametric test.

### Ethical approval

Informed consent was obtained from all the enrolled participants. The study was approved by the Ethical Committee of the National Institute for Infectious Diseases Lazzaro Spallanzani (Parere n. 271 del Registro delle Sperimentazioni, 04.02.2021) and following amendments.

Thereafter, approval was given by local ethic committees (Comitato Etico dell’Università La Sapienza, Rif n.6358; Ligurian regional ethics committee, n. CER Liguria 164/2021; Ethics Committee for Clinical Trials of the Province of Padua n. 5101/U6n/21, 17.06.2021; CE AVEC: 399/2021/Sper/AOUBo; Comitato Etico Milano Area 2 c N. 1473 del 13/05/2021).

## Results

### Study sample

The enrolment of study participants started in February 2021 and ended in September 2021. Overall, 2,497 individuals were enrolled at time of their first vaccination. The whole study sample included two main subgroups, healthy adults aged 65 years or less and frail elderly aged >65 years with two or more co-morbidities. Subjects vaccinated with mRNA vaccines and vector-based vaccines were included. Results of the first 6-month follow-up have been published previously (2). Dropout from the study during the 12-month monitoring period led to a total of 1,182 subjects available for analysis 12 months after the receipt of the first vaccine dose (T12). The demographic characteristics of the study sample are shown in **Table 1**. Most of the enrolled subjects (1055/1182) received, between T6 and T12, a third booster dose with mRNA vaccine, according to recommendations from the Italian Ministry of Health (19).

**Table 1:** Characteristics of the study sample.

VARIABLE	STRATA	NUMBER (%)	NUMBER (%)	NUMBER (%)
Group		Overall sample (Median age 55; range 18-96)	Among ≤65 years (Median age 49; range 18-65)	Among >65 years (Median age 71; range 66-96)
**Total**		1,182 (100)	875 (100)	307 (100)
**Sex**	Female	631 (53.38)	495 (56.57)	136 (44.30)
	Male	551 (46.62)	380 (43.43)	171 (55.70)
**Third dose**	Yes	1055 (89.26)	771 (88.11)	284 (92.51)
	No	127 (10.74)	104 (11.89)	23 (7.49)
**Anti-N IgG status at T12**	Positive	406 (34.35)	320 (36.57)	86 (28.01)
	Negative	650 (55.0)	441 (50.4)	209 (68.08)
	Missing	126 (10.66)	114 (13.03)	12 (3.91)
**Suppressor drugs**	Yes	36 (0.30)	1 (0.11)	35 (11.40)
	No	1146 (0.97)	874 (99.89)	272 (88.60)
**mRNA vaccines during primary cycle**	Yes	946 (80.03)	687 (78.51)	259 (84.36)
	No	236 (19.97)	188 (21.49)	48 (15.64)

### Determinants of antibody levels 12 months after first vaccination

**Figure 1:**
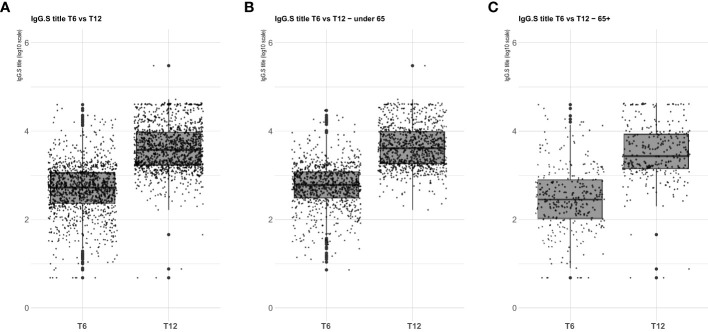
SARS-CoV-2 trimeric S IgG antibody concentration (log-transformed values) 6 months (T6) and 12 months (T12) after first dose of vaccine. Data are presented for the whole sample **(A)**, healthy adults ≤ 65 years of age (under 65, **B**), frail elderly > 65 years of age (65+, **C**). Mean values in each group are indicated (thick lines).

**Figure 2:**
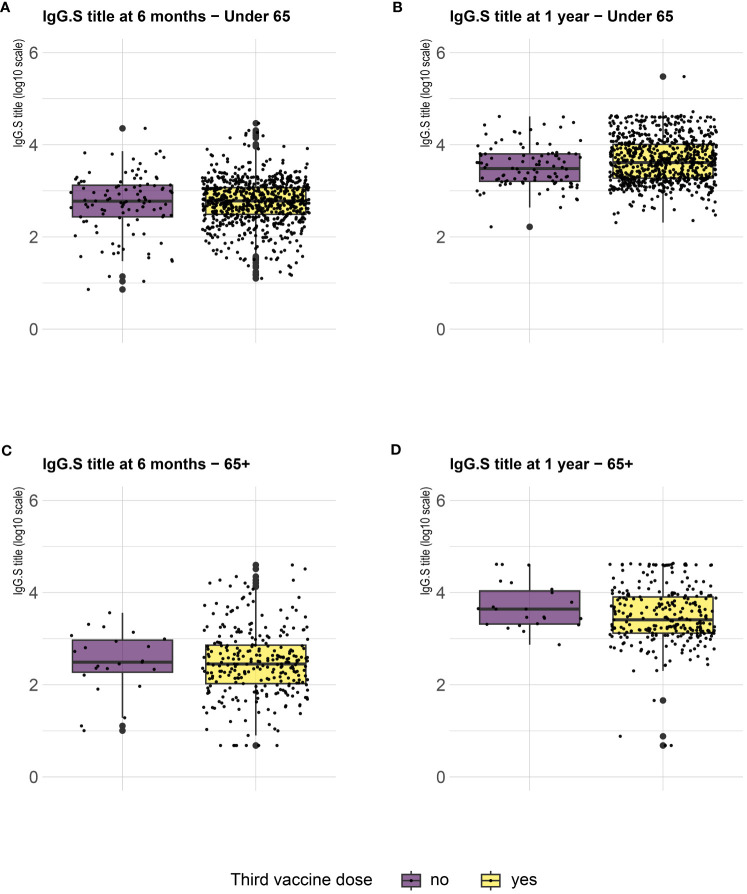
SARS-CoV-2 trimeric S IgG antibody concentration (log-transformed values) 6 months (T6) and 12 months (T12) after first dose of vaccine. Data are stratified according to the receipt of a third vaccine dose and presented for healthy adults ≤ 65 years of age (under 65, **A, B**) and frail elderly > 65 years of age (65+, **C, D**). Mean values in each group are indicated (thick lines).

**Figure 3:**
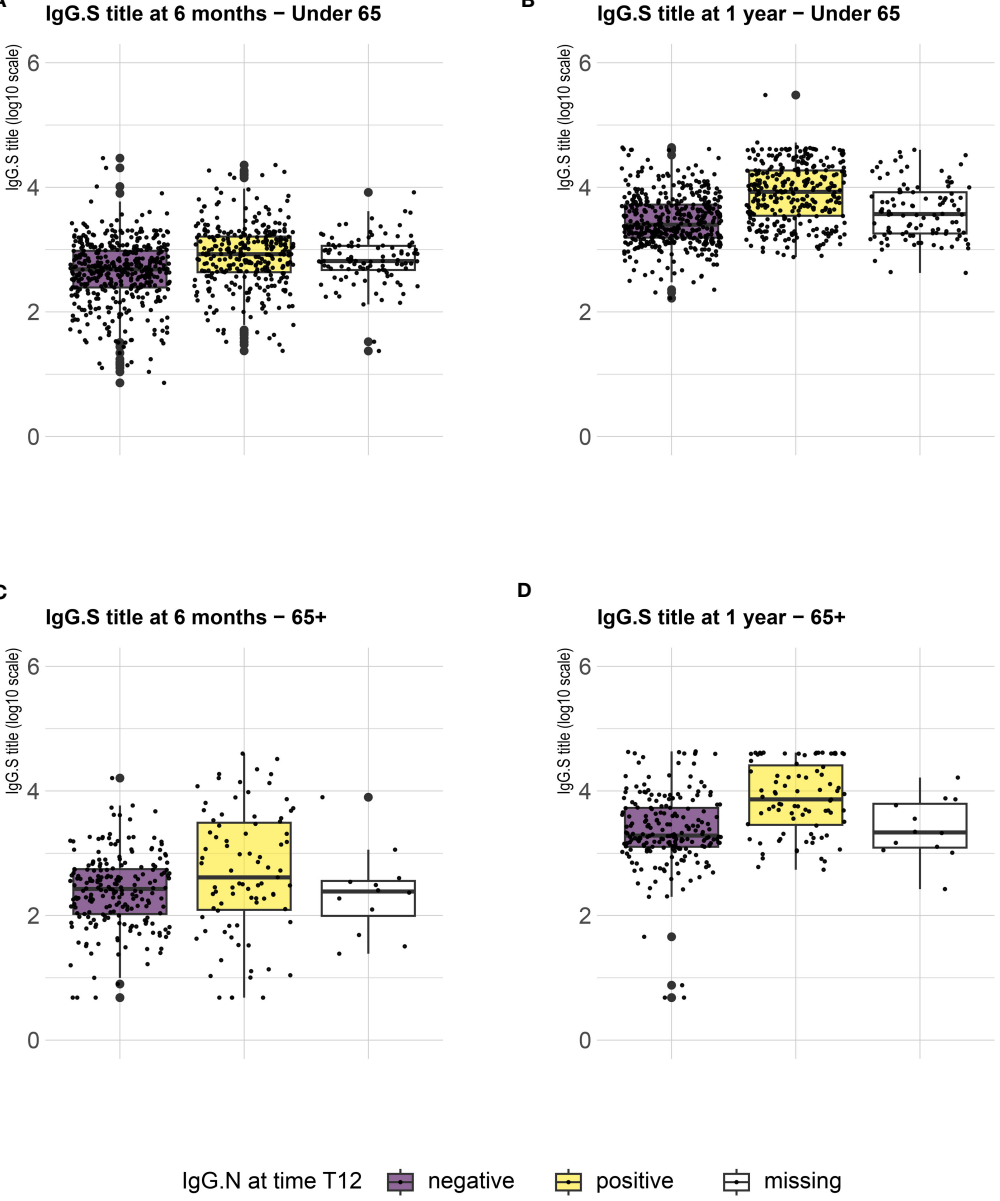
SARS-CoV-2 trimeric S IgG antibody concentration (log-transformed values) 6 months (T6) and 12 months (T12) after first dose of vaccine. Data are stratified according to anti-N IgG positivity at T12 and presented for healthy adults ≤ 65 years of age (under 65, **A, B**) and frail elderly > 65 years of age (65+, **C, D**). Mean values in each group are indicated (thick lines).

Serological analysis showed an increase of median anti-S IgG levels at T12 compared to T6, both in healthy adults and in the frail elderly group (**Figure 1**). When data were stratified according to age/comorbidities and the receipt of the third booster dose, an enhancing effect of the booster dose was noticed, both in younger and older adults, although an increase of serum anti-S IgG levels was evident also in those subjects who did not receive the third dose (**Figure 2**). When data were stratified according to positive or negative anti-N IgG test at T12, an indicator of a recent infection, it was found that anti-N IgG positive individuals displayed the higher levels of anti-S IgG levels at T12 in both age-groups (**Figure 3**).

To identify the determinants of increased serum antibody levels, we applied a log-linear model, which revealed that receipt of a third vaccine dose between T6 and T12 and positivity for anti-N IgG at T12 were strongly associated with an increase in anti-S IgG levels (**Table 2**). Males exhibited higher anti-S IgG increase compared to females (pvalue=0.018), while older frail participants showed lower antibody responses (pvalue=0.076) with respect to younger participants. When we looked in the same model at any effect according to the type of vaccine used for the primary series, we did not find any effect of using an mRNA vaccine in the primary series on the increase of antibody response.

**Table 2:** Results of the log-linear model on anti-S IgG levels at T12.

VARIABLE	Estimate	STD.ERR.	P VALUE	MEAN VARIATION (%)	95%CI (low)	95%CI (high)
**(Intercept)**	6,14087	0,18814	<0.001	–	–	–
**Third dose YES vs NO**	0,52155	0,11347	<0.001	68,5%	34,8%	110,5%
**Anti-N IgG at T3 POS vs NEG**	0,99149	0,07269	<0.001	169,5%	133,7%	210,8%
**Age group ≤65 vs >65**	0,13505	0,07614	0.076	14,5%	-1,4%	32,9%
**Log (Anti-S IgG titre-T2)**	0,17653	0,02592	<0.001	19,3%	13,4%	25,5%
**Sex M vs F**	0,15892	0,06677	0.0175	17,2%	2,8%	33,6%
**Vaccine mRNA YES vs NO**	0,0762	0,09159	0.406	7,9%	-9,8%	29,2%

### Neutralizing capacity at T12

Sera collected at T12 from a representative sample of healthy adults ≤65 years (N=39) and frail elderly individuals >65 years (N=34) who received the Comirnaty vaccine in the primary series and were COVID-19 naïve at time of their firs vaccination, were randomly selected for virus neutralization assays against the WT strain (B), and the Omicron BA.5 variant. Overall, a positive correlation was observed between anti-S IgG levels and neutralizing ability against both the ancestral B strain and the BA.5 Omicron variant (**Table 3**). Higher neutralizing capacity was observed in individuals below 65 years of age compared to those aged above 65 [B ≤65 years, median (IQR): 1024 (704-1024) *vs* B >65 years: 352 (48-1024), p=0.003; BA.5 ≤65 years, median (IQR): 416 (160-832) *vs* BA.5 >65 years: 56 (IQR: 1-416), p=0.001] Additionally, subjects who had tested positive for anti-N IgG at T12 demonstrated enhanced neutralizing ability [B ≤65 years anti-N IgG positive, median (IQR): 1024 (640-1024) *vs* B ≤65 years anti-N IgG negative: 448 (232-1024), p=0.02; B >65 years anti-N IgG positive, median (IQR): 1024 (864-1024) *vs* B >65 years anti-N IgG negative: 48 (21-768), p<0.0001; BA.5 ≤65 years anti-N IgG positive, median (IQR): 256 (168-768) *vs* BA.5 ≤65 years anti-N IgG negative: 96 (30-368), p=0.005; BA.5 >65 years anti-N IgG positive, median (IQR): 320 (96-832) *vs* BA.5 >65 years anti-N IgG negative: 1 (1-15), p<0.0001]. Sex-based differences were noted, with males exhibiting superior neutralization against the ancestral B strain (p=0.023), while no sex differences were observed for neutralizing BA.5.

**Table 3:** Spearman correlation between neutralization and anti-S IgG titre at T12.

	Rs MNT-BA/Anti-S IgG*	P VALUE	Rs MNT-B/Anti-S IgG*	P VALUE
**Overall**	0.465	0	0.468	0
**Male**	0.363	*0.045*	0.275	0.134
**Female**	0.507	*0.001*	0.535	0
**>65 years**	0.381	*0.026*	0.3	0.085
**≤65 years**	0.672	0	0.713	0
**Anti-N IgG POS**	0.585	0	0.533	0.001
**Anti-N IgG NEG**	0.361	*0.031*	0.368	0.027

*Spearman’s rank correlation coefficient of the correlation between microneutralization titers (MNT) and Anti-S IgG at T12.

**Figure 4:**
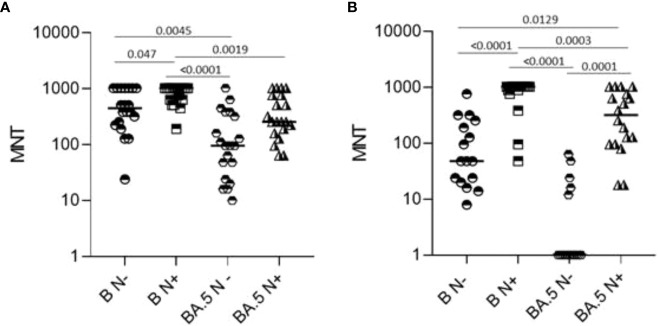
Microneutralization titers of T12 sera from healthy adults (N=39, **A**) and frail elderly subjects (N=34, panel B) who received three doses of mRNA vaccine. Data were stratified according to anti-N IgG positivity at T12 and refer to neutralization against WT SARS-CoV-2 reference strain **(B)** and a BA.5 Omicron variant strain. Individual MNT are reported together with median values. Non-neutralizing sera (cut-off MNT<8) are placed on the x-axis, Statistical differences were calculated by one-way ANOVA test,.

As shown in **Figure 4**, comparison of sera neutralizing capacity showed a higher median MNT against B as compared to BA.5. Positivity for anti-N IgG at T12 was associated with significantly higher neutralizing titers against the ancestral strain in the two age groups and against BA.5 in frail elderly individuals. Only 5/16 (31.75%) of anti-N IgG negative ≥65 subjects were able to neutralize BA.5, while anti-N IgG positive >65 subjects were all able to neutralize this strain.

### T-cell mediated immune response to vaccination

**Figure 5:**
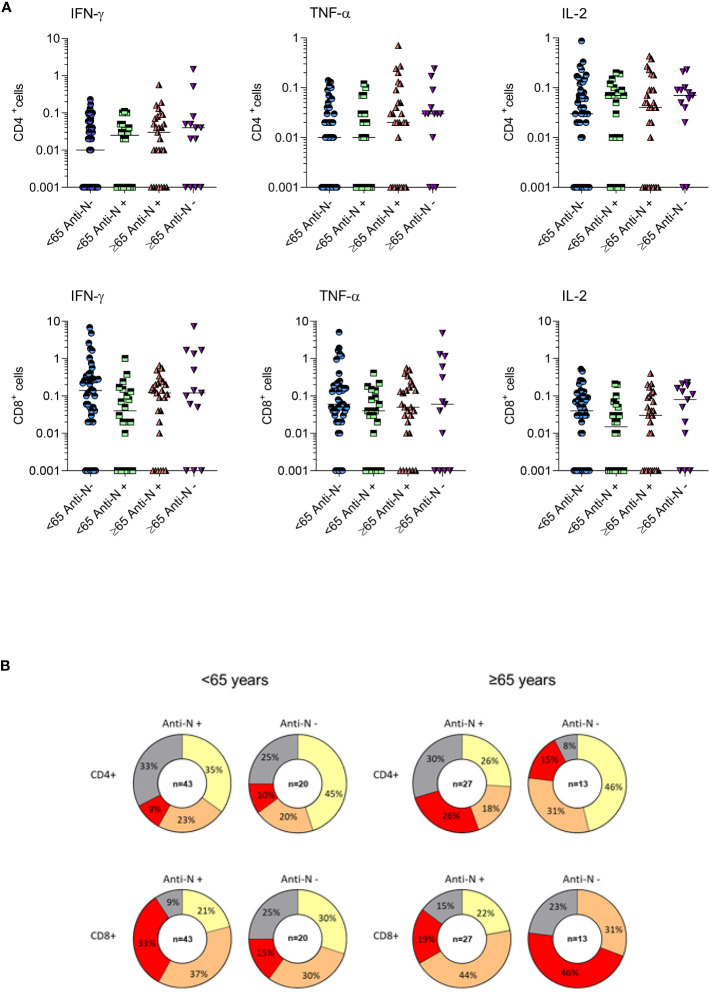
T-cell mediated immune response of COVID-19 healthy adults ≤65 years (Under, N=63) and frail elderly individuals >65 years (Over, N=40) who received three doses of mRNA vaccine. Individual frequencies of CD4+ and CD8+ T cells producing IFNγ, TNFα and IL-12 in healthy adults and frail elderly subjects in response to *in vitro* stimulation with Spike antigen are shown **(A)**. Pie diagrams show the frequencies of non-responding T cells or T cells producing from 1 to 3 simultaneous cytokines **(B)**.

The frequencies of CD4+ and CD8+ T cells producing IFN-γ, TNF-α and IL-2 in response to SARS-CoV-2 Spike peptides were measured in randomly selected COVID-19 healthy adults ≤65 years (N=63) and frail elderly individuals >65 years (N=40) who were COVID-19 naïve at time of their first vaccination and received a three-dose mRNA vaccine schedule, 12 months after the first vaccine dose. Among healthy adults, only 4/63 (6.35%) individuals did not react to Spike antigenic stimulation with the production of at least one of the three cytokines analysed (IFN-γ, TNF-α, IL-2), in CD4+ or in CD8+ T cells. Among the frail elderly subset, only 2/34 (5.88%) did not display a positive CD4+ or CD8+ T cells response to Spike. We stratified analysed individuals according to anti-N IgG positivity at T12. As shown in **Figure 5**, we found that even in the anti-N IgG negative groups very rarely the analysed subjects had a negative CMI response with no cytokine produced in response to Spike stimulation by CD4+ or CD8+ T cells (≤65: 2/43; >65: 2/27).

Comparing age groups, we found in anti-N IgG negative subjects a tendency to a preferential CD8+ vs CD4+ response in younger vs older subjects, marked by higher frequencies of polyfunctional cells producing three cytokines (polyfunctional ≤65 years: CD4 + 9%, CD8 + 33%; polyfunctional >65 years: CD4 + 26%; CD8 + 19%, not significant) In anti-N IgG positive subjects, we observed a tendency to a better response in the frail elderly group compared to healthy adults, particularly in the CD8+ compartment (polyfunctional ≤65 years: CD4 + 10%, CD8 + 15%; polyfunctional >65 years: CD4 + 15%; CD8 + 46%, not significant). When we assessed whether there was a correlation between CMI and antibody response, a positive correlation was evident between antibody titers and the frequencies of CD4+/IFNgamma+ T cells in anti-N IgG positive older subjects and between CD8+/IL-2+ T cells in anti-N IgG positive individuals, either under 65 or over 65 (**Table 4**).

**Table 4:** Spearman correlation between T-cell response and anti-S IgG titre at T12.

	≤65 years (Anti-N IgG NEG)		≤65 years (Anti-N IgG POS)			>65 years (Anti-N IgG NEG)		>65 years (Anti-N IgG POS)	
	Rs*	P VALUE	Rs	P VALUE		Rs*	P VALUE	Rs	P VALUE
**CD4 IFNγ**	-0.09	0.60	0.05	0.85	**CD4 IFNγ**	0.31	0.12	0.75	*0.007*
**CD4 IL-2**	-0.16	0.36	0.23	0.35	**CD4 IL-2**	0.14	0.47	-0.35	0.28
**CD4 TFNα**	-0.16	0.36	0.28	0.24	**CD4 TFNα**	0.29	0.13	-0.03	0.94
**CD8 IFNγ**	-0.02	0.90	0.08	0.41	**CD8 IFNγ**	-0.04	0.83	-0.09	0.79
**CD8 IL-2**	-0.02	0.90	0.59	*0.007*	**CD8 IL-2**	-0.13	0.50	-0.84	*0.002*
**CD8 TFNα**	-0.17	0.31	-0.09	0.70	**CD8 TFNα**	-0.01	0.94	-0.20	0.55

*Spearman’s rank correlation coefficient of the correlation between cytokine positive CD4/CD8 cells and Anti-S IgG at T12.

## Discussion

A real-world population-based longitudinal immune-monitoring study was designed with the aim to determine the level and durability of immunity against SARS-CoV-2. Almost 3,000 subjects were enrolled in the study cohort at time of their first SARS-CoV-2 vaccination, from February to September 2021. The study cohort was followed 12 months by estimating at fixed time-points serum anti-S and anti-N IgG, virus neutralizing activity and cell-mediated immunity. Results of the first 6-month follow-up allowed a head-to-head comparison of the immunogenicity of mRNA and vectored vaccines primary series (2). Here, we report immunological data collected at the 12-month time-point in the remaining sample of 1,182 subjects.

High levels of serum anti-S IgG were detected, antibody levels remained above the positivity threshold for the anti-S IgG assay and were increased as compared to T6. The highest increase was associated with the uptake of vaccine booster dose or with the onset of COVID-19 infection. We thus confirm that the booster dose induces an antibody response peaking at higher levels than the second dose (20–23).

In a previous report, we compared the antibody response to the four SARS-CoV-2 vaccines available in Italy in 2021 and recognized a hierarchy in anti-S IgG inducing capacity either at 1 month and 6 months after the primary vaccine series, with Spikevax > Comirnaty >Vaxzevria > Ad.26.CoV2.S (2). Worth of note, in a similar study the authors found that differences were flattened after the vaccination with a booster dose (24). Accordingly, we did not find any effect of using an mRNA vaccine in the primary series on the increase of Spike-specific antibody response.

As expected, anti-N IgG positivity at T12, an indicator of recent SARS-CoV-2 infection, was associated to higher anti-S IgG levels, pointing out the immune enhancing role of repetitive antigenic stimulation. Prior infection represents an additional immunizing event; worth of note, it has been found that, given an equal number of prior immunizing events (vaccination or previous infection), hybrid immunity results in a lower risk of infection compared to persons with vaccine-induced immunity (25, 26). Anti-N IgG seroprevalence generally paralleled COVID-19 infection, and data support the longevity of these circulating antibodies (27, 28). Our data can reinforce the utility of anti-N IgG testing as a tool to capture previous infection which, despite possibly being asymptomatic, may represent a key immunization event.

When we analysed which factors had an influence on the antibody increase observed between T6 and T12 we found that the receipt of a third vaccine dose, anti-N IgG positivity, sex, and immunosuppressant drug had a significant impact on the increase of antibody levels in the T6-T12 time frame.

Although quantitative antibody levels may be very informative, qualitative assays are crucial to understand the efficacy of vaccination. Sera neutralizing capacity was assessed at T12 against the reference strain used in the study and the Omicron BA.5 variant, as the main variant circulating in Italy at the time of T12 samples collection. The results obtained show that sera from healthy adults neutralized efficiently also the non-vaccine BA.5 strain, even when they were anti-N IgG negative, *i.e.*, in the absence of a recent infection. These data suggest that, at least in healthy adults, booster vaccination may increase the breadth of the immune response, allowing for variant neutralization, a mechanism already described and ascribed to the increased proportion of B cell clones targeting conserved regions of the receptor-binding domain (29, 30). According to the concept of “original antigenic sin”, or immune imprinting, antibody responses to secondary infections with escape mutants are dominated by specificities to the original dominating antigens. Our data support the notion that in uninfected healthy adults a functioning immune system is capable to respond to repeated vaccination increasing antibody avidity and cross-reactivity. On the contrary, not recently infected frail elderly subjects, were impaired in their ability to neutralize the ancestral Wuhan strain and only 5 out of 16 neutralized the BA.5 variant, highlighting a qualitative defect of their serological response that could be attributed, among other factors, to immunosenescence (31, 32).

Most of the analysed individuals displayed a positive CMI responses to the Spike protein, and the frequency of responders increased as compared to T6 (2). This observation is in line with humoral immune response data, showing an overall increase of anti-S immunity in the T6-T12 timeframe. An emerging consensus is growing on the protective role of T-cell immunity in reducing COVID-19 severity (33–35). Worth of note, T-cell responses against SARS-CoV-2 are not significantly disrupted by the Variant of Concern (VOC) as already reported (32, 36). This suggests that vaccine-mediated cellular cross-protection against SARS-CoV-2 VOC in older adults might compensate for evasion of neutralising antibodies by mutated Spike proteins. Interestingly, in a previous study it was found that in SARS-CoV-2 naïve nursing homes residents who responded poorly to the first immunization the administration of the booster vaccine dose restored spike-specific T-cell responses, while a previous SARS-CoV-2 infection had an impact on the magnitude of vaccine-induced cell-mediated immunity at earlier time points (37). Taken together our findings suggest the persistence of SARS-CoV-2 Spike-specific T cells following initial vaccination and the moderate effect of booster vaccination on the reactivity of these cells.

Results of the present study highlighted the vaccine potentiating effect of a recent SARS-CoV-2 infection. A high degree of these infections might be not recognized, indeed a recent systematic review showed that the combined estimate of the asymptomatic SARS-CoV-2 infection proportion is 17% (95% CI 14% to 20%) (38). Although it could be hypothesized that in the general asymptomatic SARS-CoV-2 spreading may positively impact population immunity by reducing transmission, in some settings, such as nursing homes or hospital wards asymptomatic carriers represent a significant risk for transmission. In this context, our data suggest that anti-N IgG testing might be a relevant strategy in capturing asymptomatic circulation in such settings.

The present study suffers from some limitations, such as incomplete anti-N IgG data or the lack of information on the occurrence of SARS-CoV-2 infection in vaccinated individuals. Moreover, neutralization and T-cell data were studied in subjects vaccinated with mRNA vaccines only. Another drawback is represented by the fact that these findings refer to a period in which the epidemiological context was different. Newly circulating variants are predominant and a fourth dose with bivalent mRNA vaccines directed against Omicron variants has been recommended. Nevertheless, our data drew attention on the gap that could exist between antibody levels and ability to neutralize a non-vaccine strain by frail elderly individuals who might be not fully immunocompetent, a result which should be kept in mind when implementing SARS-CoV-2 vaccines optimization and novel vaccination campaigns.

The successful deployment of COVID-19 vaccines not only relies on their initial efficacy but also necessitates a comprehensive understanding of the long-term immunological responses they induce. Immunological monitoring plays a crucial role in assessing the immune system’s response to vaccination. Insights into vaccine mechanisms, durability, and potential limitations may be gathered, ultimately informing vaccination strategies and public health interventions.

In conclusion, since we are in a transition to endemic circulation of SARS-CoV-2, monitoring the evolution of immunity to SARS-CoV-2 variants remains mandatory, with a special emphasis on monitoring the immune status of those population groups who are more at risk.

## Data availability statement

The raw data supporting the conclusions of this article will be made available by the authors, without undue reservation.

## Ethics statement

The studies involving humans were approved by Ethical Committee of the National Institute for Infectious Diseases Lazzaro Spallanzani. The studies were conducted in accordance with the local legislation and institutional requirements. The participants provided their written informed consent to participate in this study.

## Author contributions

GF: Conceptualization, Data curation, Investigation, Methodology, Writing – original draft. IS: Data curation, Formal Analysis, Methodology, Writing – review & editing. FT: Data curation, Formal Analysis, Methodology, Writing – review & editing. PL: Investigation, Writing – review & editing. EO: Investigation, Writing – review & editing. AF: Investigation, Writing – review & editing. SF: Investigation, Writing – review & editing. AD: Investigation, Writing – review & editing. SA: Conceptualization, Investigation, Writing – review & editing. VB: Conceptualization, Investigation, Writing – review & editing. TB: Conceptualization, Investigation, Writing – review & editing. AB: Conceptualization, Investigation, Writing – review & editing. PC: Conceptualization, Investigation, Writing – review & editing. MD: Conceptualization, Investigation, Writing – review & editing. FD: Conceptualization, Investigation, Writing – review & editing. AD: Conceptualization, Investigation, Writing – review & editing. FF: Conceptualization, Investigation, Writing – review & editing. IG: Conceptualization, Investigation, Writing – review & editing. AG: Conceptualization, Investigation, Writing – review & editing. RG: Conceptualization, Investigation, Writing – review & editing. TL: Conceptualization, Investigation, Writing – review & editing. VL: Conceptualization, Investigation, Writing – review & editing. CM: Conceptualization, Investigation, Writing – review & editing. RP: Conceptualization, Investigation, Writing – review & editing. VR: Conceptualization, Investigation, Writing – review & editing. FV: Conceptualization, Investigation, Writing – review & editing. SB: Supervision, Writing – review & editing. SM: Supervision, Writing – review & editing. AP: Supervision, Writing – review & editing. PS: Conceptualization, Supervision, Writing – review & editing.

## Study group for the immunological monitoring post Covid-19 vaccination:

Domenico Martinelli, Gaetano Corso, Fabio Arena, Rosella De Nittis, Giuseppina Iannelli, Alessandro Leonardi, Fabio D’Emilio, Teresa Ortuso, Policlinico Riuniti Foggia Hospital, University of Foggia, Foggia, Italy; Tiziana Coppola, Lorena Gottardello, Department of Hygiene and Public Health, Azienda ULSS 6 Euganea, Padova, Italy; Irene Amoruso, Alessandra Corazzina, Emanuela Ravazzolo, Monica Riondato, Laboratory of Hygiene and Applied Microbiology, Hygiene and Public Health Unit, Department of Cardiac Thoracic and Vascular Sciences and Public Health, University of Padova, Padova, Italy; Riccardo Boscolo, Lorenzo Carrer, Ruggero Geppini, Camilla Marcato, Marco Pinato, Claudia Scardina, Beatrice Sgorbissa, Paola Sorrentino, School of Specialization in Hygiene and Preventive Medicine, University of Padova, Padova, Italy; Guido Antonelli, Lucilla Caivano, Carolina Marzuillo, Anna Napoli, Lilia Cinti, AOU Policlinico Umberto I, Sapienza University, Rome, Italy; Francesco Vitale, Lia Caldarella, Luca Riggio, Department of Health Promotion, Mother and Child Care, Internal Medicine and Medical Specialties, University of Palermo, Palermo, Italy; Giancarlo Icardi, Andrea Orsi, Bianca Bruzzone, Valentina Ricucci, Elisabetta Costa, Matilde Ogliastro, Elvira Massaro, IRCCS Ospedale Policlinico San Martino Genova, and Department of Health Sciences, University of Genoa, Genoa, Italy; Paola Gruarin, Martina Martinovic, Serena Curti, Tanya Fabbris, Andrea Favalli, Istituto Nazionale Genetica Molecolare, Padiglione Romeo ed Enrica Invernizzi, Milan, Italy; Claudia Pavia, A;S;S;T; Ovest Milanese, Legnano, Milan, Italy; Valeria Pastore, Maria Teresa Curri, Stefano Scarpa, Antonio Muscatello, Bianca Mariani, Fondazione IRCCS Ca’ Granda Ospedale Maggiore Policlinico, Milan, Italy; Tommasina D’Elia, Chiara Gamberini, Marta Leone, Alessandro Deni, Giulia Di Felice, Paola Caffaro, Section of Microbiology, Department of Medical and Surgical Sciences, University of Bologna, Bologna, Italy; Luigina Ambrosio, Eleonora Benedetti, Department of Infectious Diseases, istituto Superiore di Sanità, Rome, Italy.
